# Genome-Wide Identification and Expression Analysis of *NF-YA* Gene Family in the Filling Stage of Wheat (*Triticum aestivum* L.)

**DOI:** 10.3390/ijms26010133

**Published:** 2024-12-27

**Authors:** Yang Zhang, Yanmin Xu, Yulu Mao, Xiaodi Tan, Yuan Tian, Xiaofei Ma, Hutai Ji, Dingyi Zhang

**Affiliations:** 1 Wheat Research Institute, Shanxi Agricultural University, Linfen 041000, China; yangzhang610@163.com (Y.Z.); xynlxyxym@163.com (Y.X.); maoyulu426@163.com (Y.M.); tanxd372557785@163.com (X.T.); 15838976678@163.com (Y.T.); zhangdingyi@sxau.edu.cn (D.Z.); 2 Agricultural College, Shanxi Agricultural University, Jinzhong 030810, China

**Keywords:** wheat, *NF-YA* gene family, expression analysis, filling stage, bioinformatics analysis

## Abstract

The *NF-YA* gene family is a highly conserved transcription factor that plays a crucial role in regulating plant growth, development, and responses to various stresses. Despite extensive studies in multiple plants, there has been a dearth of focused and systematic analysis on NF-YA genes in wheat grains. In this study, we carried out a comprehensive bioinformatics analysis of the *NF-YA* gene family in wheat, using the latest genomic data from the Chinese Spring. A total of 19 *TaNF-YA* genes were identified. An analysis of conserved domains, phylogenetic relationships, and gene structure indicated a significant degree of conservation among TaNF-YAs. A gene collinearity analysis demonstrated that fragment duplication was the predominant mechanism driving the amplification of *TaNF-YAs*. Furthermore, *cis*-acting elements within the promoters of *TaNF-YAs* were found to be implicated in grain development. Subsequently, SNP analysis revealed the genetic variation in the *NF-YA* gene family in different wheat. Moreover, published RNA-seq data were used and RNA-seqs of Pinyu8155, Yaomai30, Yaomai36, and Pinyu8175 were performed to identify *TaNF-YAs* influencing grain development. Finally, it was found that *NF-YAs* had no self-activating activity in wheat. This study provides key candidate genes for the exploration of grain development in the wheat filling stage and also lays a foundation for further research on the regulation of starch and protein synthesis and accumulation.

## 1. Introduction

Nuclear transcription factor Y (NF-Y), known as plant heme-activating protein (HAP) or CCAAT-binding factor (CBF) [1], is a heterotrimeric transcription factor complex that is composed of three subunits: NF-YA, NF-YB, and NF-YC [2]. It is prevalent across the majority of eukaryotic organisms [3]. The specific amino acid sequences within the NF-YA/B/C subunits are highly conserved throughout evolution. These sequences encompass two or three distinct protein–protein interaction domains that facilitate trimerization and interactions with other nucleoproteins [4,5]. The NF-YA domain serves as the core conserved domain of the NF-YA family, characterized by two α-helices. The N-terminal region of this domain interacts with NF-YB and NF-YC heterodimers, while the C-terminal region plays a crucial role in the recognition of DNA binding sites [6,7].

*NF-YA* was initially identified and studied in yeast and mammalian systems. It is encoded by only one or two genes [8]. With the development of genome sequencing, the *NF-YA* family has been systematically identified in several plants, including 10 *NF-YAs* in *Arabidopsis thaliana* [9], 10 *NF-YAs* in *Triticum aestivum* [10], 10 *NF-YAs* in *Oryza sativa* [11], 14 *NF-YAs* in *Zea mays* [12], 29 *NF-YAs* in *Gossypium hirsutum* [13], 9 *NF-YAs* in *Juglans regia* [14], 8 *NF-YAs* in *Vitis vinifera* [15], 21 *NF-YAs* in *Glycine max Merr* [16], and 8 *NF-YAs* in *Hordeum vulgare* [17]. Previous studies indicated that *NF-YA* genes were widely involved in plant growth processes, such as embryogenesis, seed development [18], leaf growth [19], flowering regulation [20], and responses to abiotic stressors [21,22]. At present, many studies have demonstrated that *NF-YAs* are involved in the development of plant embryos and endosperm. For instance, *TaNF-YA3/4/5/7/9* genes were preferentially expressed in the endosperm tissues of wheat [10]. In Arabidopsis, the overexpression of *NF-YA1/3/5/6/8/9* is known to regulate embryogenesis and is essential for early embryogenesis [18,23]. Additionally, *OsNF-YA8* was primarily expressed in the endosperm of monocot species [24]. *RcNF-YA2* and *RcNF-YA4* were highly expressed in the endosperm of castor seeds [25]. These findings indicate that certain *NF-YA* genes play a significant role in the development of plant grains.

Wheat (2n = 6x = 42, genome formula AABBDD) is one of the most extensively cultivated food crops in the world [26], contributing 20% of the caloric and protein intake of the human population [27]. It serves as the primary source for various grain-processing products, such as bread, biscuits, and noodles [28]. Enhancing both the yield and quality of wheat is crucial for ensuring global food security [29,30]. Grain filling represents the concluding phase of grain development, serving as a critical rate-limiting factor in the formation of grain yield [31,32]. During this phase, a large amount of starch and storage proteins are accumulated in the endosperm, which directly determines the yield and quality of wheat [33]. However, the *NF-YA* gene family has yet to be systematically identified in wheat, and the role of *NF-YAs* in the grain development of wheat has not been well studied.

In this study, we identified 19 *TaNF-YAs* in wheat utilizing a genome-wide search method, which was based on the latest available genomic information on Chinese Spring. Through bioinformatics analysis, we assessed their physical and chemical properties, gene structures, conserved domains, phylogenetic relationships, chromosome localization, collinearity, and *cis*-acting promoter elements. Furthermore, we investigated the expression profiles of *NF-YAs* in different tissues and under the abiotic stresses of wheat. Following this, we analyzed the haplotypes of *TaNF-YAs* to gain insights into their genetic variation in wheat. RNA-seq was performed on grains during the grain filling stage to identify candidate genes from the *NF-YA* gene family that influence grain development in wheat. The functional roles of *NF-YA* genes were further identified in the context of wheat.

## 2. Results

### 2.1. Conserved Domains, Phylogenetic Relationships and Chromosomal Distribution of TaNF-YAs in Wheat

**Figure 1:**
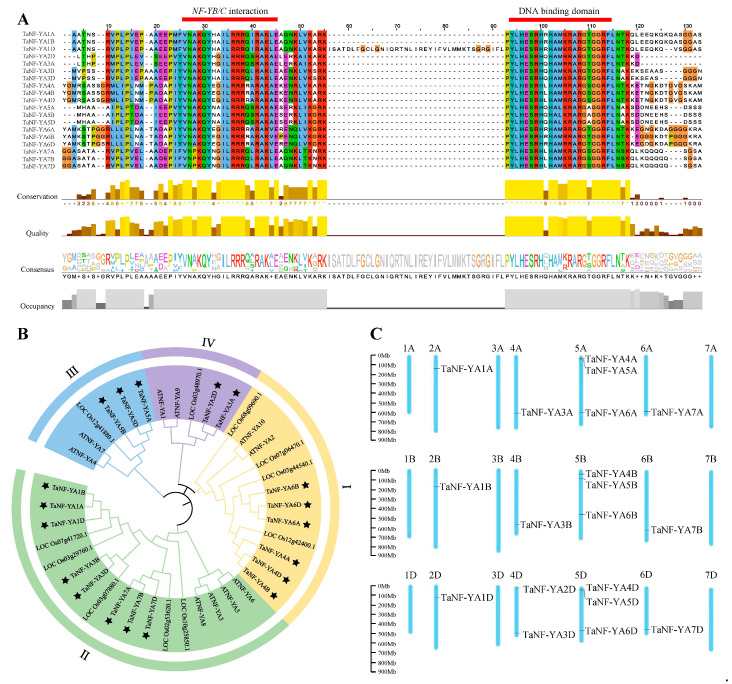
Conserved domain, evolutionary tree, and chromosome distribution of *TaNF-YA* gene family. (**A**) Sequence alignment of TaNF-YA proteins in wheat. The red rectangles represent two conserved domains of TaNF-YA. (**B**) Phylogenetic analysis of NF-YA in *Triticum aestivum*, *Oryza sativa*, and *Arabidopsis thaliana*. Yellow, green, blue, and purple branches represent subfamilies I, II, III, and IV, respectively. TaNF-YA proteins are marked with black-filled stars. (**C**) Chromosomal distribution of *TaNF-YAs* in wheat.

In this study, a total of 19 *TaNF-YAs* were identified and renamed from TaNF-YA1A to TaNF-YA7D. The conserved domain of TaNF-YAs was composed of two typical conserved domains: the NF-YB/C interaction domain and the DNA binding domain [3,34,35]. The former, located at the N-terminus, consists of 20 amino acids and facilitates protein interactions with NF-YB/C subunits, while the latter, situated at the C-terminal, consists of 21 amino acids and is responsible for specific binding to CCAAT boxes [36] (Figure 1A). The protein lengths of TaNF-YAs ranged from 207 to 341 aa. The CDS sequences ranged from 633 to 1029 bp. The molecular weights ranged from 22.28 to 37.02 kDa. The isoelectric point values ranged from 7.90 to 9.89. Predictions for subcellular localization indicated that the TaNF-YAs were predominantly located in the nucleus (Table 1).

To explore the phylogenetic relationship of NF-YAs within *Triticum aestivum*, *Oryza sativa*, and *Arabidopsis thaliana*, a neighbor-joining tree was constructed (Figure 1B, Table S1). The phylogenetic tree showed that NF-YAs were divided into four subfamilies, I, II, III, and IV, similar to classifications in previous studies [13]. The TaNF-YAs were distributed across all subfamilies. The subfamily containing the most TaNF-YA protein members was subfamily II, which contained eight TaNF-YAs. The second largest was subfamily I, which contained six TaNF-YAs. Subfamily IV was the least populous, with two TaNF-YAs. The NF-YAs of *Arabidopsis thaliana* and *Oryza sativa* were mainly distributed in subfamily II. A chromosomal localization analysis showed that 19 *TaNF-YAs* were distributed on 12 chromosomes and were unevenly distributed (Figure 1C). There were the highest on Chr5A, Chr5B, and Chr5D, which each contained three *TaNF-YAs*. Chr4D contained two *TaNF-YAs*. Chr2A, Chr2B, Chr2D, Chr4A, Chr4B, Chr6A, Chr6B, and Chr6D each contained one *TaNF-YA*.

### 2.2. Analysis of Conserved Motifs and Gene Structure of TaNF-YAs

**Figure 2:**
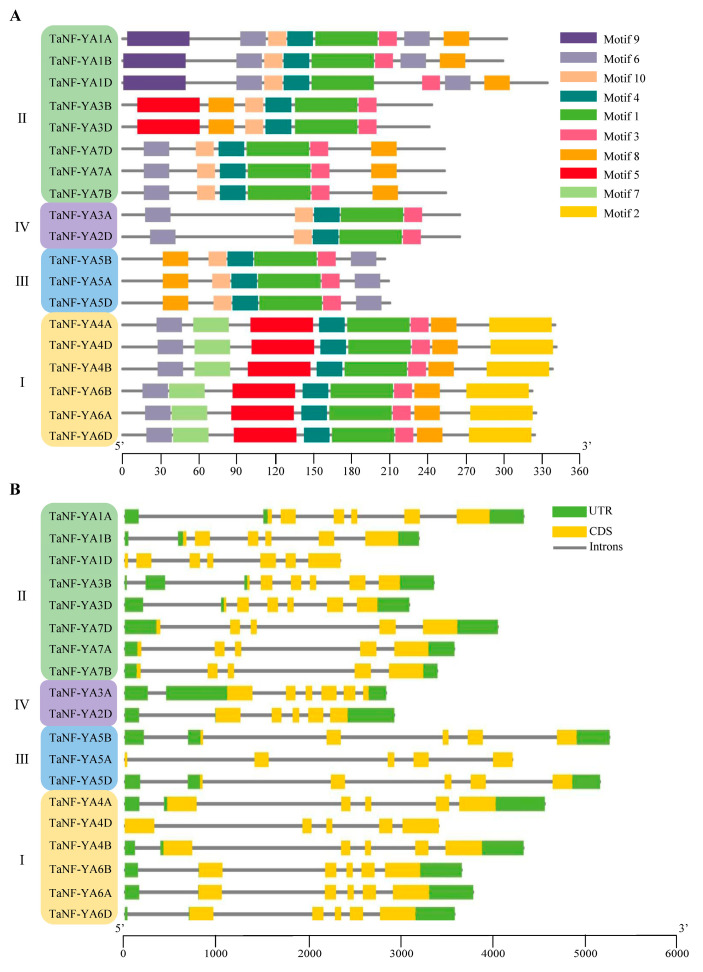
Analysis of motif and gene structure of TaNF-YAs. (**A**) Conserved motif analysis of TaNF-YAs. The different colors represent the 10 identified motifs. The motifs for each TaNF-YA are shown proportionally. (**B**) The gene structure of *TaNF-YAs*. The green and yellow rectangles represent the CDSs and UTRs, respectively. The black lines represent introns. The lengths of the CDSs, UTRs, and introns for each *TaNF-YA* are shown proportionally. I, II, III, and IV represent the different subfamilies of the *NF-YA* family.

The MEME online tool was used to analyze the conserved motifs of TaNF-YAs. The results showed that there were 10 conserved motifs in the TaNF-YAs. Motif 1 and motif 4 were widely distributed in all TaNF-YAs, indicating that they were more conserved through evolution (Figure 2A). In addition, the motif structure varied in each subfamily. For example, motif 2 and motif 7 were present only in subfamily I. Motif 8 was found in all subfamilies except subfamily IV. Motif 9 was specifically distributed in subfamily II.

A gene structure analysis showed that the *TaNF-YAs* were composed of 5–7 exons and 4–7 introns (Figure 2B). The number of exons in most *TaNF-YAs* was five. This finding was consistent with the results for *Gossypium* [13], *Cucumis sativus* [37], *Chrysanthemum morifolium* [38], and *Brassica napus* [39]. The evidence indicated that the gene structure was relatively stable and evolutionarily conserved. In addition, all *TaNF-YAs* had a complete gene structure, except for *TaNF-YA1D*, *TaNF-YA5A*, and *TaNF-YA4D*, which lacked a UTR structure.

### 2.3. Intraspecific and Intermediate Collinearity Analysis of TaNF-YAs

To further study the collinearity of the *TaNF-YAs*, the Advanced Circos plug-in for TBtools was utilized to analyze the collinearity relationship among *TaNF-YAs*. The results showed that a total of 18 *TaNF-YAs* formed 23 pairs of syntenic gene pairs, including 2 pairs of tandem duplication genes and 21 pairs of fragment duplication genes (Figure S1A), implying that fragment duplication events were the primary source for the expansion of *TaNF-YAs*. The results showed that the Ka/Ks values of all homologous gene pairs were far less than 1 (Table 2), indicating that *TaNF-YAs* have undergone strong purifying selection during evolution.

**Figure 3:**
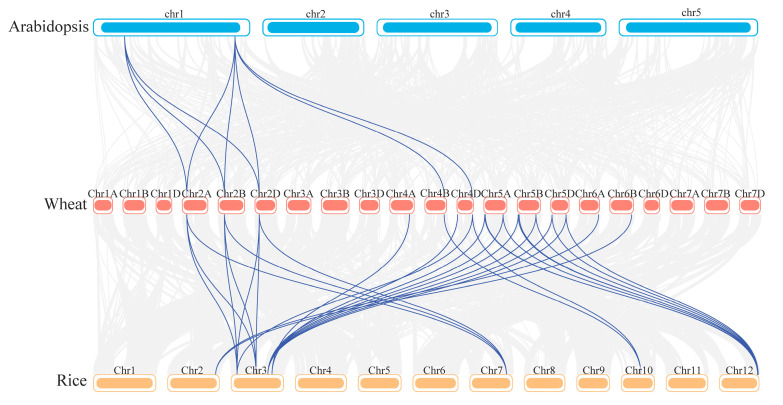
Synteny analysis related to *NF-YAs* between the genomes of wheat, Arabidopsis, and rice. The light gray lines represent the collinear blocks in wheat vs. Arabidopsis and wheat vs. rice. The syntenic *TaNF-YAs* pairs are shown connected by blue lines.

To further explore the interspecific evolutionary relationship of *NF-YAs*, the collinearity of Arabidopsis, rice, and wheat was analyzed using the Multiple Synteny Plot plug-in of TBtools. The results showed that the *NF-YAs* of Arabidopsis, rice, and wheat showed collinearity (Figure 3). There were a total of 8 collinearity genes pairs between wheat and Arabidopsis, while there were 30 pairs of collinearity genes between wheat and rice.

### 2.4. Analysis of cis-Acting Elements in the Promoter of TaNF-YAs in Wheat

**Figure 4:**
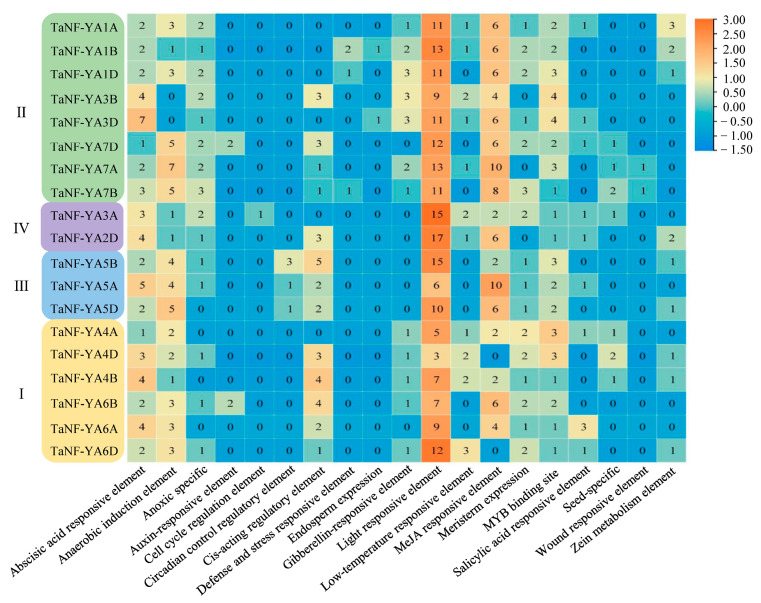
*Cis*-acting element analysis of *TaNF-YAs*. *TaNF-YAs* are listed on the Y axis; yellow, green, blue, and purple represent different subfamilies of the *TaNF-YA* family. All *cis*-elements in promoter regions are listed on the X-axis. The figure indicates the number of *cis*-elements. I, II, III, and IV represent the different subfamilies of the *NF-YA* family.

To better understand the transcriptional regulation and potential function of *TaNF-YAs*, we used the Plant CARE database to predict the 2000 bp cis-acting regulatory elements upstream of *TaNF-YA* promoters. In total, 19 functional *cis*-elements were obtained (Figure 4). Ten *TaNF-YAs* have endosperm expression elements (GCN4_motif) [40] or seed-specific cis-acting elements (RY-element) [41], indicating that these genes might participate in grain development. Additionally, abscisic acid-responsive elements, light-responsive elements, and MYB binding sites were found in all *TaNF-YAs*, and light-responsive elements account for the largest proportion of all elements.

To preliminarily predict the biological function of TaNF-YA genes, gene ontology (GO) analysis was first performed to annotate them into 168 GO terms, including 143 biological process terms (BP), 18 cell component terms (CC), and 7 molecular function terms (MF) (Table S2). BP, CC, and MF accounted for 85.1%, 10.7%, and 4.2% of the total GO items, respectively (Figure S1B).

### 2.5. Haplotype Analysis of NF-YAs in Wheat

To further explore the genetic variation of *TaNF-YAs*, a haplotype analysis was performed on 19 *TaNF-YAs* with genetic variation information using Lufei resequencing data. As shown in Table 3, there was no genetic variation in *TaNF-YA3D/4B/7D*, indicating that these genes were relatively stable and had better adaptability to different environments. Only 16 genes were found to have genetic variation information. Of the 16 TaNF-YAs, only 7 genes were found to have major haplotypes (*TaNF-YA1D/2D/4A/4D/5D/6A/6D*) (Tables S3–S5). Among them, *TaNF-YA1D/4A/4D/5D/6D* showed the same primary haplotype in the corresponding cultivated varieties, landrace varieties, spelt haplotype distribution, haplotype distribution in different continents, and spring and winter wheat haplotype distribution. The major haplotypes of *TaNF-YA2D/6A* were the same in the corresponding cultivar, the landrace haplotype distribution, as well as spring and winter wheat, while different major haplotypes were found in spelt and each continent. Other genes showed different haplotypes in different classifications.

### 2.6. Analysis of TaNF-YAs Expression Pattern Based on RNA-Seq

**Figure 5:**
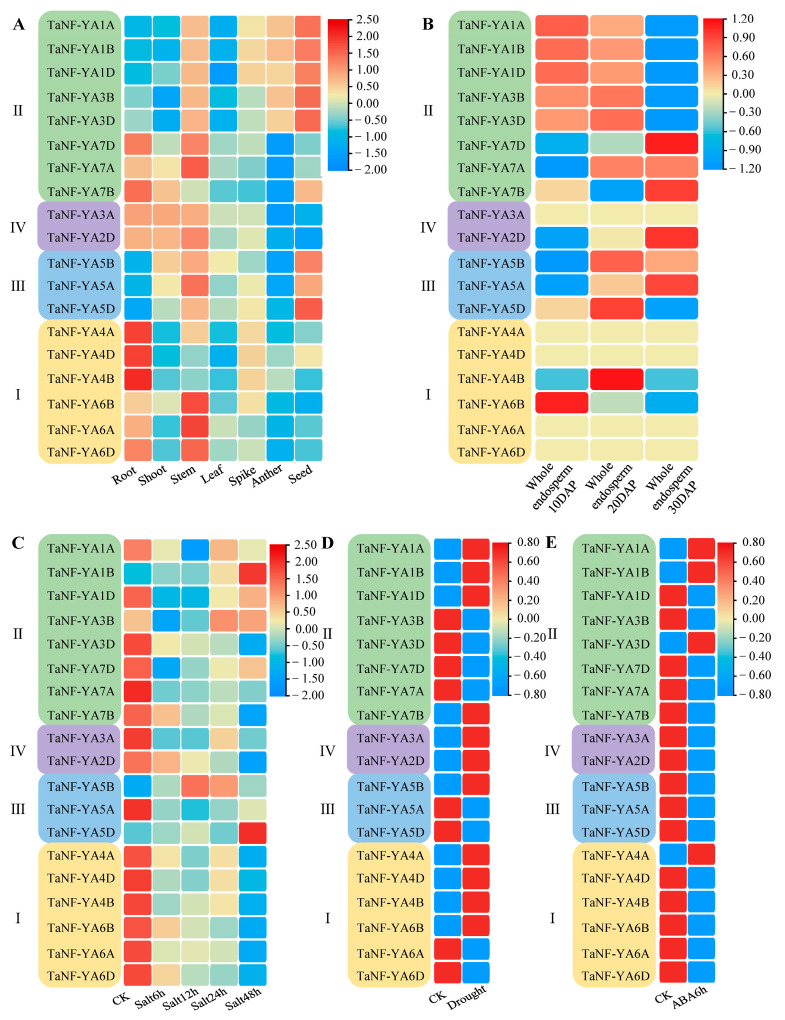
Expression patterns of *TaNF-YAs*. (**A**) A heatmap of *TaNF-YAs* in root, stem, leaf, seed, seeding, shoot, spike, and anther. (**B**) Analysis of expression patterns of *TaNF-YAs* in endosperm on different days after pollination. (**C**–**E**) Heatmap of *TaNF-YA* response to salt, drought, and ABA stress, respectively. I, II, III, and IV represent the different subfamilies of the *NF-YA* family.

The tissue-specific expression profiles, grain development, salt, drought, and ABA stress of *TaNF-YAs* were studied using the published RNA-seq data (Table S6). The results showed that the expressions of *TaNF-YAs* in the different tissues of wheat had strong tissue specificity (Figure 5A). For example, almost all the *TaNF-YAs* were expressed in the stem, but the expression level was low in the leaf. The *TaNF-YAs* of subfamily I were highly expressed in roots but had a low expression in seeds. The *TaNF-YAs* of subfamily III were highly expressed in seeds but had a low expression in roots.

To further understand the function of *TaNF-YAs* in grain development, the endosperm of grains on different days after pollination was analyzed. The results showed that most *TaNF-YAs* were expressed during grain development, with differences in expression levels, except for some genes that were not expressed (Figure 5B). For example, *TaNF-YAs* in subfamily I and subfamily III were expressed, but genes in subfamily II and subfamily IV were not expressed, except *TaNF-YA2D*, *TaNF-YA4B*, and *TaNF-YA6B*.

Finally, the expression patterns of *TaNF-YAs* under diverse abiotic stresses were also investigated based on the published RNA-seq data. The results showed that *TaNF-YAs* were expressed in salt stress, drought stress, and ABA stress. For example, *TaNF-YA1B* and *TaNF-YA5D* had an up-regulated expression after 48 h of salt stress (Figure 5C). Most *TaNF-YAs* were up-regulated under drought stress (Figure 5D). The expression of *TaNF-YA1A*, *TaNF-YA1B*, *TaNF-YA3D*, and *TaNF-YA4A* was up-regulated at 6 h under ABA stress (Figure 5E). This also confirms the accuracy of promoter *cis*-acting element prediction.

**Figure 6:**
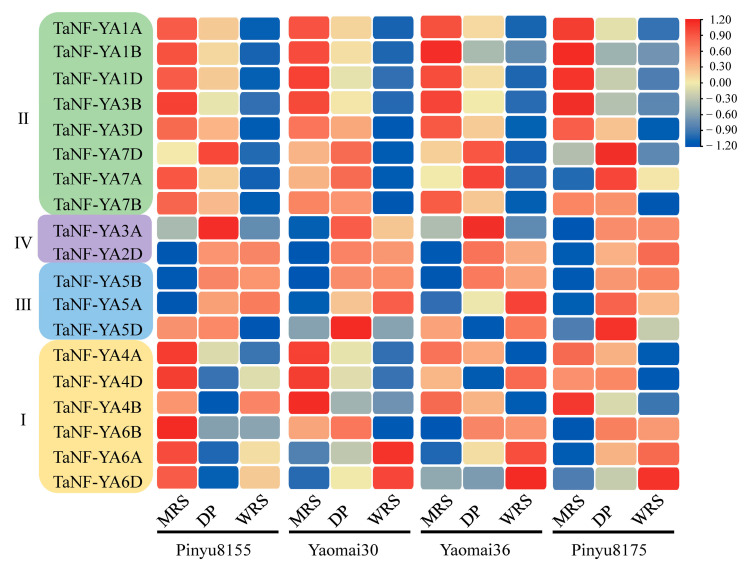
RNA-seq analysis of *TaNF-YAs* in grain development. Grain expression heatmap of Pinyu8155, Yaomai30, Yaomai36, and Pinyu8175 at filling stage. MRS. milk ripe stage, DP. dough period, WRS. wax ripe stage. I, II, III, and IV represent the different subfamilies of the *NF-YA* family.

Based on the prediction results of TaNF-YA genes on wheat grain development, an RNA-seq of TaNF-YA genes was performed at the grain filling stage of Pinyu8155, Yaomai30, Yaomai36, and Pinyu8175 (Figure S2 and Table S7). In Pinyu8155, 13 *TaNF-YAs* were found to be differentially expressed (Table 4). *TaNF-YA1A/1B/1D/3B/3D/4A/4D/6D/7B* were highly expressed at the milk ripe stage, *TaNF-YA5D/7D* were highly expressed at the dough period, and *TaNF-YA2D/5A* were highly expressed at the wax ripe period (Figure 6). In Yaomai30, 18 *TaNF-YAs* were found to be differentially expressed (Table 4). *TaNF-YA1A/1B/1D/3B/3D/4A/4B/4D* were highly expressed at the milk ripe stage, *TaNF-YA2D/3A/5B/6B/7A/7D* were highly expressed at the dough period, and *TaNF-YA5A/6A/6D* were highly expressed at the wax ripe stage (Figure 6). In Yaomai36, nine *TaNF-YAs* were found to be differentially expressed (Table 4). *TaNF-YA1A/1B/1D/2D/3B/4B/7D* were highly expressed at the milk ripe stage, *TaNF-YA5B* was highly expressed at the dough period, and *TaNF-YA5A* was highly expressed at the wax ripe stage (Figure 6). In Pinyu8175, 12 *TaNF-YAs* were found to be differentially expressed (Table 4). *TaNF-YA1A/1B/1D/3B/3D* were highly expressed at the milk ripe stage, *TaNF-YA4D/5A/7A/7D* were highly expressed at the dough period, and *TaNF-YA2D/5B/6D* were highly expressed at the wax ripe stage (Figure 6).

### 2.7. Expression Level Detection and Autoactivating Activity Verification

**Figure 7:**
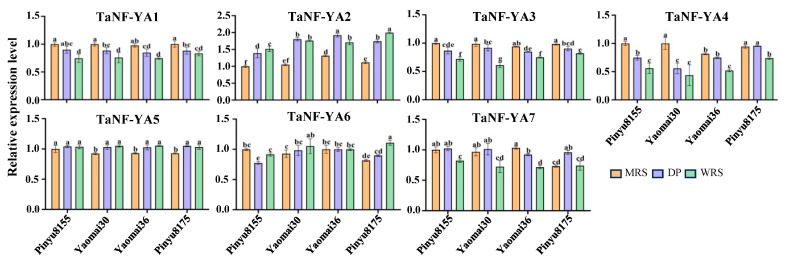
qRT-PCR analysis of *TaNF-YAs* in grain development. *TaActin* was used as a reference gene. Each error bar represents the standard deviation of three biological replicates. MRS. milk ripe stage, DP. dough period, WRS. wax ripe stage. Different lowercase letters indicate a statistical difference at *p* < 0.05 level.

**Figure 8:**
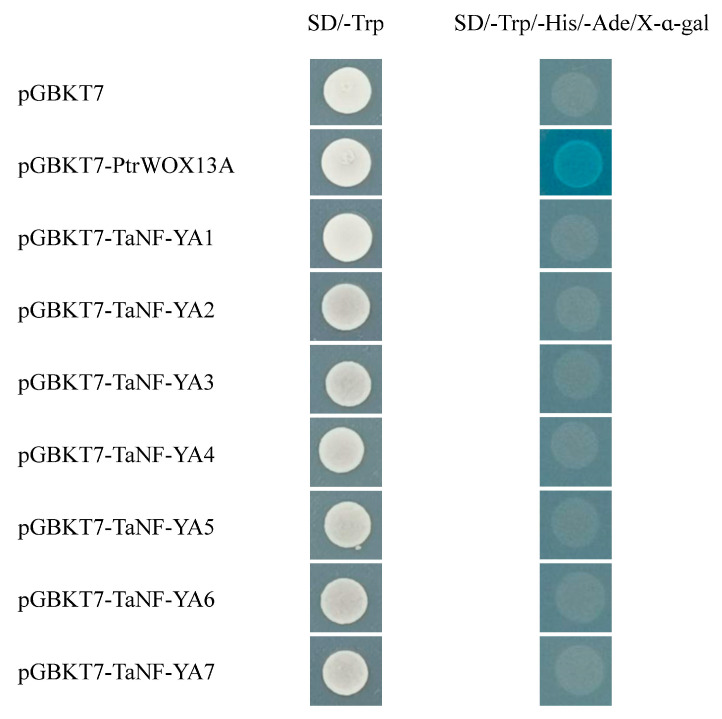
Transcriptional activation assay of TaNF-YAs.

In Figure 7, the results show that *TaNF-YAs* were expressed during the development of wheat grains, and the expression trends aligned with those observed in the RNA-seq data. There was no significant difference in the expression levels of *TaNF-YA5* among the four varieties. The expression patterns of *TaNF-YA1/3/4* exhibited similarities across all four varieties, with a gradual decrease in their expression levels. In Pinyu8155, the expression levels of *TaNF-YA6* initially decreased before increasing, while in Yaomai30 and Pinyu8175, these levels increased gradually, and in Yaomai36, there was no significant change. The expression levels of *TaNF-YA7* followed a similar trend in Pinyu8155, Yaomai30, and Pinyu8175, initially increased before decreasing, while a gradual decrease was noted for Yaomai36. For *TaNF-YA2*, the expression levels in Pinyu8155 and Pinyu8175 gradually increased, while in Yaomai30 and Yaomai36, it first increased before decreasing. This indicates that *TaNF-YA2/5/6/7* were specifically expressed in four varieties. All *TaNF-YAs* were tested for autoactivating activity in yeast, and the results were consistent with negative controls, indicating none of these genes possessed autoactivating activity (Figure 8).

## 3. Discussion

*NF-YA* is a conserved transcription factor in plants that plays a significant regulatory role in their growth and development [13]. Based on the genome-wide search method, we identified 19 Ta*NF-YAs* in wheat. A bioinformatic analysis revealed that all TaNF-YAs possess the typical domain of the NF-YA family (PF02045) (Figure 1A). These TaNF-YAs could be further divided into four groups (Figure 1B), which is consistent with the classification results of *Arabidopsis thaliana* and *Oryza sativa* [9,11]. The results showed that the TaNF-YA proteins in wheat presented the highest similarity with the proteins of *Oryza sativa* (Figure 1B). TaNF-YAs within the same subfamily share analogous gene structures and conserved motifs, indicating that these genes might perform similar biological functions (Figure 2).The collinearity relationship between *Arabidopsis thaliana* and wheat also indicates that the *NF-YAs* have undergone amplification in wheat and are functionally differentiated to adapt to complex environments [8] (Figure 3). Fragmentation and tandem duplication are important mechanisms for generating and amplifying plant gene families [42]. *TaNF-YAs* exhibit 2 pairs of tandem duplications and 21 pairs of fragment duplications, indicating that fragment duplication is the primary mode of amplification for *TaNF-YAs* (Figure S1A). However, previous studies by Stephenson et al. identified only 10 *TaNF-YA*s in wheat [10], This discrepancy might be attributed to our use of the latest database of Chinese Spring (IWGSC v2.1) and newer software versions, while Stephenson et al. used the Chinese Spring (IWGSC v1.1) database.

### 3.1. Functional Prediction of TaNF-YAs in Grain Development

Recently, *TaNF-YAs* have been identified in many plant species, including Arabidopsis [9] and rice [11]. Previous studies have demonstrated that *AtNF-YA1/3/5/6/8/9* and *OsNF-YA8* play a role in regulating embryonic development [18,23,24]. In this study, *AtNF-YA3/5/6/8* and *OsNF-YA8* (*LOC os10g15850.1)* were classified within the same subfamily as *TaNF-YA1A/1B/1D/3B/3D/7A/7B/7D* (Figure 1B). Additionally, *AtNF-YA1/9* and *TaNF-YA2D/3A* were also found in the same subfamily (Figure 1B). These genes within the same subfamily might exhibit analogous biological functions and play a significant role in grain development. However, RNA-seq and qRT-PCR results revealed that a greater number of genes were expressed during the development of wheat grains, such as *TaNF-YA4A/4B/4D/5A/5B/5D/6A/6B/6D*. This might be due to the complex and large genome of wheat and its long life cycle. Compared with diploid plants like Arabidopsis and rice, the population effect of wheat grain development is large, and the key genes are not easy to excavate [43,44]. Consequently, it is necessary to delve deeper into the key genes in the process of the grain development of wheat.

Promoter *cis*-elements are crucial for the initiation of gene expression [45]. We found that only two genes were involved in endosperm expression (Figure 4), but RNA-seq and qRT-PCR results showed that the majority of the genes were expressed during grain development. This discrepancy might arise from the fact that the prediction of promoter elements was based on the background of Chinese Spring, while the RNA-seq data were derived from winter wheat samples. Furthermore, there might be some elements related to grain development in the promoter that have not been predicted. The prediction of the *TaNF-YA* profile showed that *TaNF-YAs* had a strong tissue expression specificity (Figure 5A), and the up-regulated genes during grain development were mainly distributed in subfamilies II, III, and IV (Figure 5B). Following this, we performed a gene ontology analysis, revealing that the functions associated with GO:0010262, GO:0009790, GO:0009567, GO:0010154, GO:0009791, GO:0048316, GO:0048506, and GO:0048509 were linked to grain development, with the enriched genes primarily situated within subfamilies II, III, and IV (Figure S1B). This further verified the accuracy of the grain expression pattern prediction.

### 3.2. RNA-Seq Analysis of TaNF-YAs Among Different Varieties

It is generally believed that wheat quality is a complex trait that is usually negatively correlated with yield [46]. Yaomai36 is classified as a strong-gluten wheat, whereas Pinyu8175 is categorized as a medium-gluten wheat (Table S7). Between them, the 1000-grain weight and grain length of Pinyu8175 were significantly higher than those of Yaomai36 (Figure S2). *TaNF-YA4B/7B* were only expressed in Yaomai36, while *TaNF-YA3D/4D/5B/6D/7A/7D* were only expressed in Pinyu8175 (Table 4). Studies have shown that 1000-grain weight is a complex trait controlled by multiple genes, with genetic parameters such as grain length and width serving as important indicators for assessing wheat grain quality [47]. This indicates that *TaNF-YA4B/7B* might play a role in regulating the grain filling stages of strong-gluten wheat, while *TaNF-YA3D/4D/5B/6D/7A/7D* might regulate the grain filling stage of medium-gluten wheat.

Pinyu8155 belongs to dryland wheat, and Yaomai30 belongs to irrigated wheat, with both varieties classified as medium–strong gluten wheat (Table S7). The 1000-grain weight, grain length, and grain diameter of Yaomai30 were significantly superior to those of Pinyu8155 (Figure S2). *TaNF-YA5D* was exclusively expressed in the grain of Pinyu8155, while *TaNF-YA3A/4B/5B/6A/6B/7A* were only expressed in Yaomai30 (Table 4). Compared with Yaomai30, Pinyu8155 exhibited a smaller grain morphology and fewer expressed genes in the grains. It was speculated that some genes in Pinyu8155 might be involved in the regulation of irrigated wheat. *TaNF-YA5D* might be a key gene regulating grain development in dryland wheat, while *TaNF-YA3A/4B/5B/6A/6B/7A* might be the key gene regulating grain development in water-saving regulation.

However, we found that there was a difference between the RNA-seq results (Figure 6) and the predicted results of grain expression patterns (Figure 5B). For example, *TaNF-YA3A/4A/4D/6A/6D* were not expressed in the grains of Chinese Spring, yet RNA-seq data showed that *TaNF-YA3A* was expressed during the middle and late stages of grain filling across four varieties. *TaNF-YA4A/4D* were expressed in the early stage of grain filling. *TaNF-YA6A/6D* were expressed in the early stage of grain filling of Pinyu8155, while they were expressed in the late stage of grain filling of the other three varieties. This discrepancy might be due to the former being based on winter wheat as the experimental material, while the latter is based on the prediction of Chinese Spring as the background. Due to the wide variety of wheat varieties and the large genome, the same genes might exhibit significant differences between different wheat varieties [48]. In addition, the filling period of wheat is as long as one month [49], during which the change in meteorological factors is also an important factor affecting gene expression [50].

RNA-seq results showed that the expression levels of *TaNF-YAs* were different during the grain filling stage (Figure 6). We hypothesize that these different genes might directly or indirectly influence the synthesis and accumulation of starch and protein in grain [51]. Currently, the excavation of its regulatory genes is in progress.

### 3.3. Haplotype Analysis of TaNF-YAs

SNPs are the most abundant type of sequence variation in plant genomes [52]. A haplotype analysis of genotypes can not only elucidate the genetic differences between various materials but also provide the opportunity to identify superior allelic variations that can contribute to breeding [53]. To adapt to the complex and mutable geographical environment, wheat exhibits significant genetic variation and a large number of haplotypes. The results of this study showed that most of the genes with major haplotypes were consistent in breeding selection, geographical distribution, and spring and winter wheat distribution, indicating that these haplotypes were relatively stable, more popular in breeding selection, and more favorable to the survival of wheat in different environments. Therefore, these haplotypes were selected and fixed by breeders to participate in the growth and development of wheat. The haplotypes of genes without major haplotypes differed across various classifications, and the number of haplotypes was large, indicating that these haplotypes have evolved in different environments to adapt to complex environments and thus participate in the growth and development of wheat.

## 4. Materials and Methods

### 4.1. Plant Materials

The Pinyu8155, Yaomai30, Yaomai36, and Pinyu8175 wheat varieties were sourced from the Wheat Research Institute at Shanxi Agricultural University. These varieties were cultivated at the Hancun Experimental Base of the Wheat Research Institute of Shanxi Agricultural University (36°13.2′ N, 111°33.7′ E) during the 2023 to 2024 growing season. Each variety was planted in an area measuring 36 m in length and 10 m in width. Wheat ears that flowered on the same day were marked, and seeds were collected at three distinct stages, the milk ripe stage (about 10 days after flowering, MRS), the dough period (about 20 days after flowering, DP), and the wax ripe stage (about 25 days after flowering, WRS) during the filling stage. The collected samples were immediately frozen in liquid nitrogen and stored at −80 °C for subsequent RNA-Seq and qRT-PCR analyses.

### 4.2. Genome-Wide Identification of NF-YA Genes in Wheat

Firstly, the genome information for Chinese Spring (IWGSC v2.1) was downloaded from the JGI website (https://phytozome-next.jgi.doe.gov/, (accessed on 19 March 2024)) by searching for the target species within the Chinese Spring database [28,54]. The Arabidopsis protein sequences for NF-YA were obtained from the *Arabidopsis thaliana* database TAIR10 (https://www.arabidopsis.org/, (accessed on 21 March 2024)) [55]. The typical domain (PF02045) of NF-YA was obtained through NCBI BLAST (https://blast.ncbi.nlm.nih.gov/Blast.cgi, (accessed on 23 March 2024)) [56] and downloaded from the PFAM database [57] (https://pfam.xfam.org/, (accessed on 26 March 2024)) to construct a Hidden Markov Model (HMM). Meanwhile, the HMM search function in HMMER3.0 [58] was utilized to obtain *NF-YAs* and filter the results (E-value < 0.01). *NF-YA* transcripts were sourced from the Phytozome website (https://phytozome.jgi.doe.gov/pz/portal.html, (accessed on 1 April 2024)) [59]. Redundant transcripts of identical genes were deleted, retaining only the primary transcript. Finally, *NF-YAs* of wheat have been obtained. The protein sequences of NF-YAs were retrieved from the Chinese Spring protein database using TBtools 1.115 software [60]. The original data were optimized to obtain the protein sequences of NF-YAs. The basic physical and chemical properties, including amino acid length (bp), molecular weight (Mv), and isoelectric point (pI), of NF-YAs were analyzed using the ExPASy-Prot Param website (https://web.expasy.org/protparam/, (accessed on 3 April 2024)) [61]. The Phytozome website (https://phytozome.jgi.doe.gov/pz/portal.html, (accessed on 12 April 2024)) was used to find the location of the gene chromosome. The subcellular localization of NF-YAs was predicted using the Plant-mPLoc server website [62] (http://www.csbio.sjtu.edu.cn/bioinf/plant-multi/#, (accessed on 18 April 2024)).

### 4.3. Conserved Domains, Phylogenetic Relationships, and Chromosomal Distribution

Multiple sequence alignments of TaNF-YAs were performed using MEGA 11 software [63], with the results visualized through Jalview 2.11.3.2 [64]. NF-YA protein sequences of *Oryza sativa* and *Arabidopsis thaliana* were obtained from the PlantTFDB website [65] (http://planttfdb.gao-lab.org/index.php, (accessed on 22 April 2024)) and screened. Using the ClustalW program within MEGA, multiple sequence alignments of NF-YAs in *Oryza sativa*, *Arabidopsis thaliana*, and wheat were performed. A phylogenetic tree was constructed using the neighbor-joining (NJ) method with a bootstrap value of 1000 [66]. A visual analysis of the phylogenetic tree was performed using the iTOL website [67] (https://itol.embl.de, (accessed on 6 August 2024)), along with classification of the tree [13,68]. The chromosomal localization of *TaNF-YA*s was visualized utilizing the TBtools software.

### 4.4. Gene Structure and Conserved Motifs Analysis of TaNF-YAs

The MEME analysis website (https://meme-suite.org/meme/tools/meme, (accessed on 14 August 2024)) was used to submit the NF-YA protein sequence to obtain the MEME file, and the maximum number of motifs retrieved was 10 [69]. The Gene Structure View plug-in in TBtools was utilized to draw a diagram illustrating the intron–exon structure and create a motif diagram of the genes.

### 4.5. Collinearity Analysis of TaNF-YAs

A collinearity analysis of *TaNF-YAs* was performed and a Circos diagram generated using the Multiple Collinear Scan Toolkit X (MCScanX) plug-in in TBtools [70]. The coding sequences (CDS) of *TaNF-YAs* were aligned using the Basic Local Alignment Search Tool (BLAST) online website (https://blast.ncbi.nlm.nih.gov/Blast.cgi, (accessed on 3 September 2024)) in NCBI [71]. The gene pairs, protein sequences, and CDS sequences of *NF-YA* were used to calculate the nonsynonymous substitution rate (Ka), synonymous (Ks) substitution rate, and Ka/Ks ratio through the Ka/Ks_Calculator plug-in in the TBtools software [72]. The homology of gene pairs was analyzed using BLAST (https://blast.ncbi.nlm.nih.gov/Blast.cgi, (accessed on 7 September 2024). The collinearity between wheat, rice, and Arabidopsis was analyzed and visualized with the Multiple Synteny Plot plug-in of TBtools.

### 4.6. Analysis of cis-Acting Elements of TaNF-YAs in Wheat

The 2 000 bp sequence upstream of the CDS of *TaNF-YAs* was obtained using TBtools software and submitted to the Plant CARE [73] (https://bioinformatics.psb.ugent.be/webtools/plantcare/html/, (accessed on 1 September 2024)) website for promoter *cis*-acting element prediction. The Basic Biosequence View plug-in in TBtools was used to visualize the predicted *cis*-acting elements.

### 4.7. GO Enrichment and Haplotype Analysis of TaNF-YAs

The agriGO v2.0 website [74] (http://systemsbiology.cau.edu.cn/agriGOv2/, (accessed on 3 September 2024)) was utilized to perform a singular enrichment analysis on the members of the *TaNF-YAs*. The SNPs of *TaNF-YAs* were extracted according to the chromosomal positions of genes by using the whole genome resequencing data of wheat from LuFei (http://wheat.cau.edu.cn/WheatUnion/b_4/, (accessed on 13 September 2024)) [75]. The classification of haplotypes for re-sequenced wheat materials was based on SNP differences.

### 4.8. Gene Expression Analysis of Wheat

The publicly available RNA-seq data were downloaded from the *Triticum aestivum* RNA-seq Database website (http://ipf.sustech.edu.cn/pub/wheatrna/, (accessed on 5 October 2024)) to obtain FPKM values, to study the expression patterns in wheat. Grains at the grain filling stage were sent to Beijing Qingke Biotechnology Co., Ltd. (Beijing, China) for transcriptome sequencing. The expression heatmap of *TaNF-YAs* was generated using TBtools.

### 4.9. qRT-PCR Analysis

RNA of the filling stage in Pinyu8155, Yaomai30, Yaomai36, and Pinyu8175 was used for synthesizing cDNA. Samples of cDNA were run in triplicate using the Applied Biosystems 7500 Real-Time PCR System to determine the critical threshold (Ct) with the SYBR premix ExTaq kit (TaKaRa). The primers used for the analysis of the expression levels of the *TaNF-YAs* by qRT-PCR are listed in Table S8. *TaActin* was employed as the internal control, and the delta–delta CT method was used to quantify gene expression levels relative to *TaActin* as described in the previous report [76].

### 4.10. Transcriptional Activation Assay

The transcriptional activation of TaNF-YA on putative targets was corroborated using the yeast two-hybrid system. The complete CDS of *TaNF-YAs* was amplified using specifically designed primers (Table S8). The amplified fragments were fused in-frame to the pGBKT7 vector to generate the pGBKT7-TaNF-YAs construct. The pGBKT7-TaNF-YAs pGBKT7-PtrWOX13A (as positive control), and pGBKT7 blank vector (as negative control) were transformed into Y2H yeast cells independently. The transformed Y2H yeast cells were plated onto SD/-Trp (growth control), SD/-Trp/-His/-Ade, and X-α-gal media and incubated at 30 °C for 3–5 days to identify the transcriptional activation [76].

### 4.11. Statistical Analysis

Dunnett’s test (SPSS 20.0) was used to test the statistical significance of data. Differences between the two groups of data for comparisons in this study were evaluated by statistical significance (0.01 < *p* < 0.05).

## 5. Conclusions

In summary, *TaNF-YAs* play a crucial role in plant development and stress responses. However, their specific functions in wheat grain development necessitate further investigation. We identified 19 *TaNF-YAs* and analyzed the characteristics of these gene family members through various approaches, including conserved domains, phylogenetic relationships, gene structures, conserved motifs, collinearity, promoter *cis*-acting elements, haplotypes, and expression patterns. Furthermore, a comprehensive bioinformatics analysis combined with RNA-seq data revealed that the *TaNF-YAs* exhibited distinct expression patterns during the grain filling stage. This indicates that the NF-YA gene family is involved in regulating the grain development process. These findings provide a foundation for a deeper understanding of the molecular mechanisms underlying grain development in crops.

## Figures and Tables

**Table 1:** Basic information of *NF-YAs* identified in wheat.

Gene Name	Gene ID	Chr	Start	End	CDS Length (bp)	Amino Acid Length (aa)	MW (KDa)	pI	Subcellular Location
*TaNF-YA1A*	TraesCS2A03G0358600	2A	133,675,481	133,679,820	912	303	33.08	9.62	Nucleus
*TaNF-YA1B*	TraesCS2B03G0478600	2B	184,491,589	184,494,788	903	300	32.80	9.70	Nucleus
*TaNF-YA1D*	TraesCS2D03G0374600	2D	126,142,858	126,145,203	1008	335	36.70	9.62	Nucleus
*TaNF-YA2D*	TraesCS4D03G0056200	4D	15,856,361	15,859,292	801	266	28.70	9.15	Nucleus
*TaNF-YA3A*	TraesCS4A03G0713900	4A	584,263,143	584,265,986	801	266	28.72	9.41	Nucleus
*TaNF-YA3B*	TraesCS4B03G0764200	4B	574,599,438	574,602,800	735	244	26.56	9.34	Nucleus
*TaNF-YA3D*	TraesCS4D03G0686200	4D	460,899,556	460,902,650	729	242	26.41	9.32	Nucleus
*TaNF-YA4A*	TraesCS5A03G0073100	5A	27,789,570	27,794,138	1026	341	37.01	8.86	Nucleus
*TaNF-YA4B*	TraesCS5B03G0067200	5B	27,706,506	27,710,843	1020	339	36.77	8.86	Nucleus
*TaNF-YA4D*	TraesCS5D03G0096600	5D	37,108,522	37,111,936	1029	342	37.02	8.65	Nucleus
*TaNF-YA5A*	TraesCS5A03G0110100	5A	41,592,229	41,596,445	633	210	22.58	7.90	Nucleus
*TaNF-YA5B*	TraesCS5B03G0121600	5B	54,106,639	54,111,911	624	207	22.28	7.90	Nucleus
*TaNF-YA5D*	TraesCS5D03G0131000	5D	50,555,036	50,560,204	636	211	22.74	7.90	Nucleus
*TaNF-YA6A*	TraesCS5A03G0896800	5A	573,400,560	573,404,349	981	326	35.06	9.20	Nucleus
*TaNF-YA6B*	TraesCS5B03G0942800	5B	556,202,392	556,206,057	972	323	34.61	8.81	Nucleus
*TaNF-YA6D*	TraesCS5D03G0858600	5D	455,426,682	455,430,270	978	325	34.84	9.20	Nucleus
*TaNF-YA7A*	TraesCS6A03G0864300	6A	570,722,413	570,725,998	765	254	24.53	9.89	Nucleus
*TaNF-YA7B*	TraesCS6B03G1035100	6B	647,043,462	647,046,862	768	255	27.70	9.89	Nucleus
*TaNF-YA7D*	TraesCS6D03G0733500	6D	445,208,290	445,212,347	765	254	27.70	9.77	Nucleus

**Table 2:** Ka/Ks ratio and homology of homologous genes.

Homologous Genes		Ka	Ks	Ka/Ks	Homologous Fragment Length	Identities (%)
Gene 1	Gene 2					
*TaNF-YA1A*	*TaNF-YA1B*	0.02368	0.06350	0.37283	900	95.51
*TaNF-YA1A*	*TaNF-YA1D*	0.03145	0.06904	0.45557	894	96.52
*TaNF-YA1B*	*TaNF-YA1D*	0.02228	0.04906	0.45409	894	97.25
*TaNF-YA2D*	*TaNF-YA3A*	0.02788	0.20110	0.13865	783	90.88
*TaNF-YA3B*	*TaNF-YA3D*	0.00730	0.13063	0.05586	726	94.70
*TaNF-YA4A*	*TaNF-YA4B*	0.00916	0.09089	0.10081	1014	94.51
*TaNF-YA4A*	*TaNF-YA4D*	0.00647	0.09925	0.06524	1023	97.08
*TaNF-YA4A*	*TaNF-YA6D*	0.21782	1.01595	0.21440	951	84.38
*TaNF-YA4B*	*TaNF-YA4D*	0.00521	0.06768	0.07705	1017	97.28
*TaNF-YA4B*	*TaNF-YA6D*	0.21387	1.00565	0.21267	948	84.38
*TaNF-YA4D*	*TaNF-YA6D*	0.22150	0.99874	0.22178	951	84.38
*TaNF-YA5A*	*TaNF-YA5B*	0.01386	0.11561	0.11988	618	94.79
*TaNF-YA5A*	*TaNF-YA5D*	0.00417	0.10015	0.04166	630	97.01
*TaNF-YA5B*	*TaNF-YA5D*	0.01807	0.08383	0.21561	621	92.92
*TaNF-YA6A*	*TaNF-YA6B*	0.02831	0.09301	0.30438	960	92.15
*TaNF-YA6A*	*TaNF-YA6D*	0.02667	0.12945	0.20605	969	93.27
*TaNF-YA6B*	*TaNF-YA6D*	0.02111	0.13037	0.16191	963	93.42
*TaNF-YA7A*	*TaNF-YA7B*	0.00698	0.09714	0.07190	762	94.89

**Table 3:** Haplotype number of *TaNF-YAs*.

Gene ID	Variety			Geographical Location Distribution					Variety Type		
	Spelt	Cultivate	Landrace	Asia	Africa	Europe	N_ America	S_ America	Facultative	Spring	Winter
*TaNF-YA1A*	13	6	13	27	5	17	5	2	3	21	20
*TaNF-YA1B*	13	9	21	33	3	21	3	3	8	19	27
*TaNF-YA1D*	4	1	9	11	2	5	1	2	3	8	6
*TaNF-YA2D*	6	2	5	10	4	7	2	1	1	9	7
*TaNF-YA3A*	12	2	9	22	4	12	1	1	4	13	15
*TaNF-YA3B*	14	6	11	17	3	20	1	2	2	15	21
*TaNF-YA3D*	0	0	0	0	0	0	0	0	0	0	0
*TaNF-YA4A*	3	2	4	4	1	4	1	2	3	2	4
*TaNF-YA4B*	0	0	0	0	0	0	0	0	0	0	0
*TaNF-YA4D*	7	3	6	15	4	8	1	3	3	11	10
*TaNF-YA5A*	14	8	25	41	5	22	2	2	6	25	29
*TaNF-YA5B*	14	8	34	46	6	21	2	2	7	36	28
*TaNF-YA5D*	4	1	2	2	1	4	2	1	1	3	3
*TaNF-YA6A*	7	6	9	12	4	9	2	2	3	9	11
*TaNF-YA6B*	14	8	20	37	5	18	4	2	4	28	22
*TaNF-YA6D*	8	1	3	14	5	8	1	1	3	9	7
*TaNF-YA7A*	14	7	30	44	6	24	3	2	5	30	34
*TaNF-YA7B*	14	7	18	30	5	22	4	2	6	21	23
*TaNF-YA7D*	0	0	0	0	0	0	0	0	0	0	0

**Table 4:** Differently expressed genes associated with grain development in the filling stage.

Variety	Gene Name	FPKM Mean			*p*-Value	Level
		MRS	DP	WRS		
Pinyu8155	*TaNF-YA1A*	9.2427	8.2509	6.6084	1.42397 × 10^−54^	significant
	*TaNF-YA1B*	8.6953	7.6101	6.2446	8.06098 × 10^−57^	significant
	*TaNF-YA1D*	9.4130	8.7314	7.5454	1.23465 × 10^−18^	significant
	*TaNF-YA2D*	3.2953	4.5944	4.6723	0.019459322	significant
	*TaNF-YA3B*	8.6177	7.5035	6.6598	2.68707 × 10^−17^	significant
	*TaNF-YA3D*	9.0269	8.2087	6.0066	4.35495 × 10^−34^	significant
	*TaNF-YA4A*	4.2471	2.8896	2.1787	4.23058 × 10^−8^	significant
	*TaNF-YA4D*	4.5815	3.5064	3.9296	0.000959117	significant
	*TaNF-YA5A*	6.8201	7.5547	7.6498	2.63508 × 10^−6^	significant
	*TaNF-YA5D*	7.6664	7.6808	7.2214	0.006655729	significant
	*TaNF-Y6D*	4.9625	3.8850	4.5690	0.003850238	significant
	*TaNF-YA7B*	4.9012	4.6018	3.8650	0.002255128	significant
	*TaNF-YA7D*	4.4480	4.9347	3.9810	0.002752711	significant
Yaomai30	*TaNF-YA1A*	9.2266	8.0759	6.5569	3.4975 × 10^−68^	significant
	*TaNF-YA1B*	8.6308	7.4663	6.2025	1.64548 × 10^−50^	significant
	*TaNF-YA1D*	9.2164	8.4105	7.7947	5.4985 × 10^−16^	significant
	*TaNF-YA2D*	3.5246	5.9733	5.7686	2.31801 × 10^−15^	significant
	*TaNF-YA3A*	4.3450	5.2354	4.9273	0.018687011	significant
	*TaNF-YA3B*	8.5723	7.5461	6.5612	2.79623 × 10^−16^	significant
	*TaNF-YA3D*	9.1852	8.4993	5.6980	2.01475 × 10^−40^	significant
	*TaNF-YA4A*	4.0146	2.1491	1.1342	1.46941 × 10^−11^	significant
	*TaNF-YA4B*	4.3123	2.9474	2.7664	0.000618621	significant
	*TaNF-YA4D*	4.9266	4.3011	3.8881	0.006868434	significant
	*TaNF-YA5A*	6.6612	7.1964	7.4600	0.000297905	significant
	*TaNF-YA5B*	6.8550	7.7639	7.7562	3.74578 × 10^−9^	significant
	*TaNF-YA6A*	4.4237	4.6709	5.3353	0.002324020	significant
	*TaNF-YA6B*	4.3938	4.5630	3.5285	0.009245228	significant
	*TaNF-YA6D*	4.9368	5.3232	5.7216	0.004492247	significant
	*TaNF-YA7A*	4.8932	5.2123	3.9293	0.000295765	significant
	*TaNF-YA7B*	4.3957	4.3197	3.0679	0.003992178	significant
	*TaNF-YA7D*	4.3183	4.7026	3.1900	0.000811669	significant
Yaomai36	*TaNF-YA1A*	9.0306	7.9856	6.7145	0.000365685	significant
	*TaNF-YA1B*	8.5750	7.0378	6.7101	2.82713 × 10^−10^	significant
	*TaNF-YA1D*	9.1267	8.1578	7.0790	2.44115 × 10^−5^	significant
	*TaNF-YA2D*	4.2185	6.1064	5.7250	5.07876 × 10^−5^	significant
	*TaNF-YA3B*	8.2840	7.4122	6.6357	0.000300512	significant
	*TaNF-YA4B*	3.6900	3.3183	2.2875	0.002718470	significant
	*TaNF-YA5A*	6.7630	7.1355	7.5710	0.013510010	significant
	*TaNF-YA5B*	6.8289	7.7378	7.6101	0.003405333	significant
	*TaNF-YA7B*	4.9223	4.3562	3.3867	0.000261033	significant
Pinyu8175	*TaNF-YA1A*	9.1541	8.1787	7.4869	3.00122 × 10^−17^	significant
	*TaNF-YA1B*	8.5854	7.4209	7.2649	1.74623 × 10^−24^	significant
	*TaNF-YA1D*	9.6246	8.5066	8.0277	1.28126 × 10^−15^	significant
	*TaNF-YA2D*	3.6950	5.7659	6.5086	2.54353 × 10^−14^	significant
	*TaNF-YA3B*	8.5155	7.6455	7.3909	1.86229 × 10^−6^	significant
	*TaNF-YA3D*	8.8771	8.3205	7.2108	6.22932 × 10^−10^	significant
	*TaNF-YA4D*	4.1775	4.2057	3.2935	0.016155841	significant
	*TaNF-YA5A*	6.7540	7.5032	7.2890	0.000185483	significant
	*TaNF-YA5B*	6.7807	7.7076	7.7768	9.91354 × 10^−11^	significant
	*TaNF-YA6D*	4.0213	4.4202	5.4000	0.000200123	significant
	*TaNF-YA7A*	3.3347	4.3805	3.8482	0.016966263	significant
	*TaNF-YA7D*	3.4529	4.6553	3.1579	0.000111526	significant

## Data Availability

Data are contained within the article and Supplementary Materials.

## Supplementary Materials

The following supporting information can be downloaded at: https://www.mdpi.com/article/10.3390/ijms26010133/s1.
