# Supplementary material for: Genome-Wide Identification and Expression Analysis of NF-YA Gene Family in the Filling Stage of Wheat (Triticum aestivum L.)
Source: Int J Mol Sci. 2024 Dec 27;26(1):133. doi: 10.3390/ijms26010133 (PMC11719562; doi:10.3390/ijms26010133)
Supplement: Supplementary file 1 [file ijms-26-00133-s001.zip › Figure S.pdf]

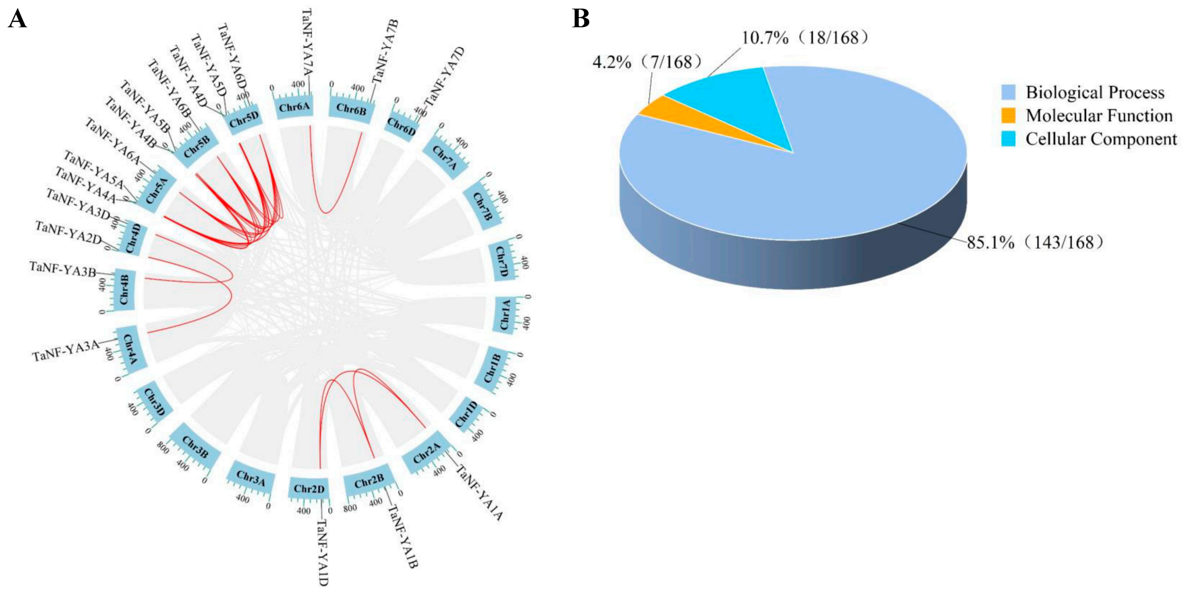

Figure S1. Collinearity analysis and Gene Ontology analysis of *TaNF-YA* gene family. A. The collinearity analysis of *TaNF-YAs*. The gray lines represent the synteny blocks in wheat among chromosomes, duplicated *TaNF-YAs* gene pairs are shown by red lines. The twenty-one chromosomes are displayed on the map in a circular pattern. B. The proportion of GO items of Biological Process, Cellular Component, and Molecular Function.

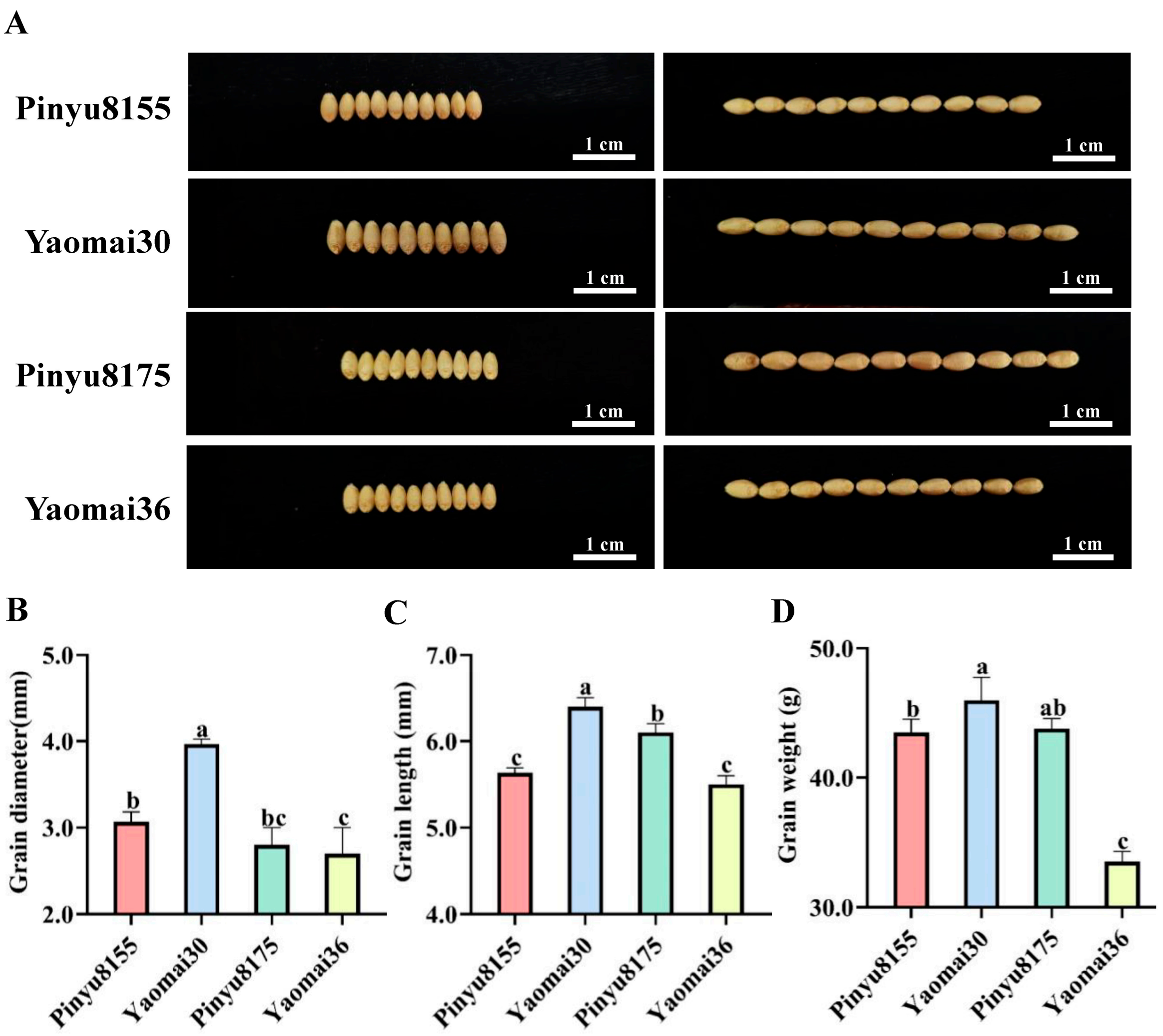

Figure S2. The grain morphology of 4 varieties. A. Picture of seed development of four varieties. B. Grain diameter size of four varieties. C. Grain length of four varieties. D. Grain weight of four varieties.
